# The problem of home choice in skyline-based homing

**DOI:** 10.1371/journal.pone.0194070

**Published:** 2018-03-09

**Authors:** Martin M. Müller, Olivier J. N. Bertrand, Dario Differt, Martin Egelhaaf

**Affiliations:** 1 Department of Neurobiology, Faculty of Biology, and Cluster of Excellence ‘Cognitive Interaction Technology’ (CITEC), Bielefeld University, Bielefeld, Germany; 2 Computer Engineering Group, Faculty of Technology, Bielefeld University, Bielefeld, Germany; University of Sussex, UNITED KINGDOM

## Abstract

Navigation in cluttered environments is an important challenge for animals and robots alike and has been the subject of many studies trying to explain and mimic animal navigational abilities. However, the question of selecting an appropriate home location has, so far, received only little attention. This is surprising, since the choice of a home location might greatly influence an animal’s navigation performance. To address the question of home choice in cluttered environments, a systematic analysis of homing trajectories was performed by computer simulations using a skyline-based local homing method. Our analysis reveals that homing performance strongly depends on the location of the home in the environment. Furthermore, it appears that by assessing homing success in the immediate vicinity of the home, an animal might be able to predict its overall success in returning to it from within a much larger area.

## Introduction

### Home choice, an understudied problem?

The ability to return to a known location, in the following called homing, is a vital problem to many animals and has been extensively studied, especially in insects (e.g.: [1–9]). However, while much work has been put into the analysis of visual homing behaviours in insects and other animals, most studies focus on the conditions under which different strategies allow for successful homing to an already established home location and the comparison of different homing algorithms. In contrast to this, much less is known about whether the choice of a home location itself might enable a navigator to return to its home more successfully.

From a biological perspective, the potential importance of home choice can be illustrated by the example of a bumblebee queen in spring, trying to find a suitable place to start a new colony and build her nest. Once she has chosen a location and laid the first eggs, she will have to feed the newly hatched larvae of the first workers. To accomplish this, repeated foraging trips are necessary [10, 11]. If the queen ever fails to return to the newly founded nest, her brood will be lost. Thus, the ease of homing to a potential nest location is of utmost importance to the queen. The same applies to the foragers who will later take over the task of supplying the colony. This argument can be extended to any central place foragers, like ants, bees, wasps, or birds looking to find a suitable place for a new nest, or more generally any animal which repeatedly needs to visit a specific location.

Surprisingly however, very little is known about the processes underlying the initial selection of a proper home location from a navigational perspective. Although Richards [12] performed an investigation into nest site selection of bumblebees, this analysis has focused mostly on ecological parameters and does not address navigational issues. Janson et al. [13] looked into nest choice in honey bees, but focused on the decentralised decision making process and not the choice itself. This is remarkable, since the choice of the location of one’s home might play a crucial role for successful navigation.

Therefore, this study will address the question whether the choice of the home location matters, and if so, whether homing performance can be improved by selecting a suitable home location.

### Visually guided navigation

One of the first formalised models, used to explain the astonishing navigational feats of honey-bees, is the snapshot model proposed by Cartwright and Collett [14]. In their original model, the current panorama seen by an animal is compared with a “snapshot” taken at the goal location (e.g. a flower or the bee’s nest). This “snapshot” consists of the pattern of light and dark areas on the retina seen at a given location, as was most congruent with experimental results obtained for honey-bees by Cartwright and Collett. Using these patterns to segment the image, the relative displacement between corresponding image segments can be used to generate a new heading direction leading back to the target (“homing direction”).

Since then, many different methods have been proposed to explain and mimic the visually guided navigation abilities of insects (amongst the most notable being gradient descent on image differences [6, 15], recognition-triggered response strategies [16, 17], or scene familiarity methods [18], which recently have also been implemented in a mushroom-body inspired network architecture [19]).

In this study we focus on a variant of the Average Landmark Vector (ALV) model, a long established model of snapshot-based local homing [20]. Here, a panorama is reduced to a vector representing the average bearing of all detected features (called “landmarks”), like edges or dark patches in the image. The homing direction is then derived by simply subtracting the ALV determined at the home location, from the one computed at the current location of the agent. The ALV model is very interesting from the perspective of insect navigation, due to its simplicity regarding computational and memory requirements and its compatibility with the retinotopic architecture of the insect visual system [21].

**Fig 1:**
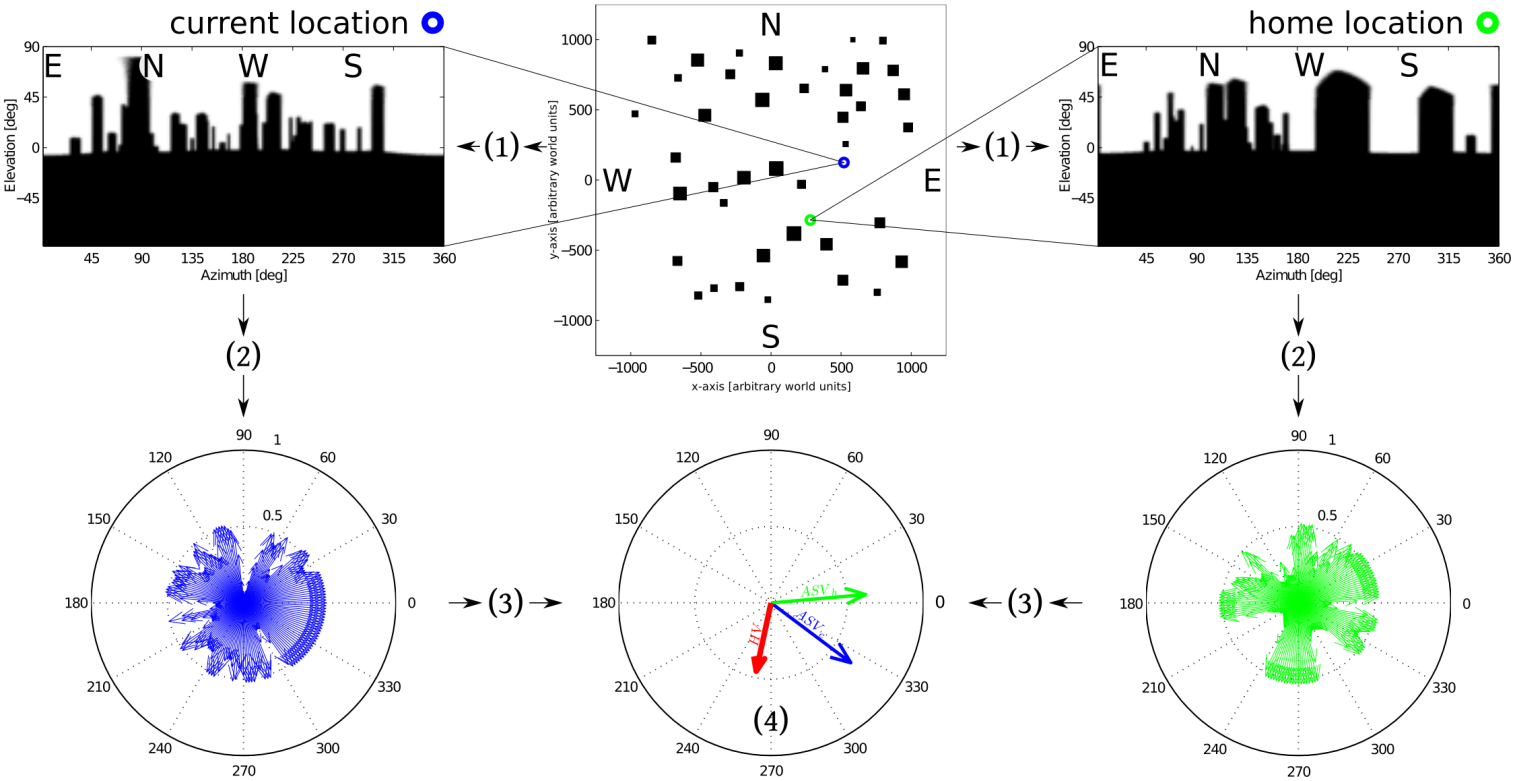
Overview of the ASV homing method. **Top row**: The *central panel* shows a top view of the simulated cluttered environment. Black boxes represent objects, the **blue** and **green** markers indicate the current and home location of the agent. To perform a homing run between these two locations, panoramic views are rendered (1) at each location (*left and right panel*) using the CyberInsect toolbox [36]. **Bottom row**: Vector profiles of each scene are created (2) taking the given viewing direction as each vector’s argument and the relative pixel intensity of the corresponding image column as its norm. This represents the skyline of each scene (*left and right panel*). Summing (3) these vector profiles yields the Average Skyline Vector (ASV) of each scene *(central panel*, **blue** and **green** arrows). (4) Subtracting the ASVs yields the homing vector (HV, *central panel*, **red** arrow). The agent will follow this HV for a given distance until another HV is generated at its new location.

However, since extracting features is a computationally demanding task, especially in cluttered, naturalistic environments, the model has been adapted to use low-level characteristics, like pixel intensity values, without further processing [21–23]. In one such variant, termed the Centre of Mass Average Landmark Vector model, the ALV of a scene corresponds to the centre of mass of its pixel intensity values [22–25]. To this end, intensity values in each image column are first averaged along the elevation of the image. If the measured intensity values of the ground are consistently lower than those of the sky, this corresponds to extracting the skyline of a given black and white panorama. We will thus call this variant of the ALV the Average Skyline Vector (ASV) model (see Fig 1 for an overview of the ASV algorithm and Methods for a formal description). Such a skyline-based mechanism is very appealing from an insect perspective, because the skyline is invariant under illumination changes as caused by changing lighting and weather conditions [26, 27]. Several experimental studies suggest that insects might base their navigational endeavours on skyline cues [28–30], while multiple technical studies could establish the feasibility of this approach both experimentally [29, 31, 32] and theoretically [33–35]. We will thus use the ASV model to systematically investigate the question of home choice.

### Linking catchment area size and environmental characteristics

**Fig 2:**
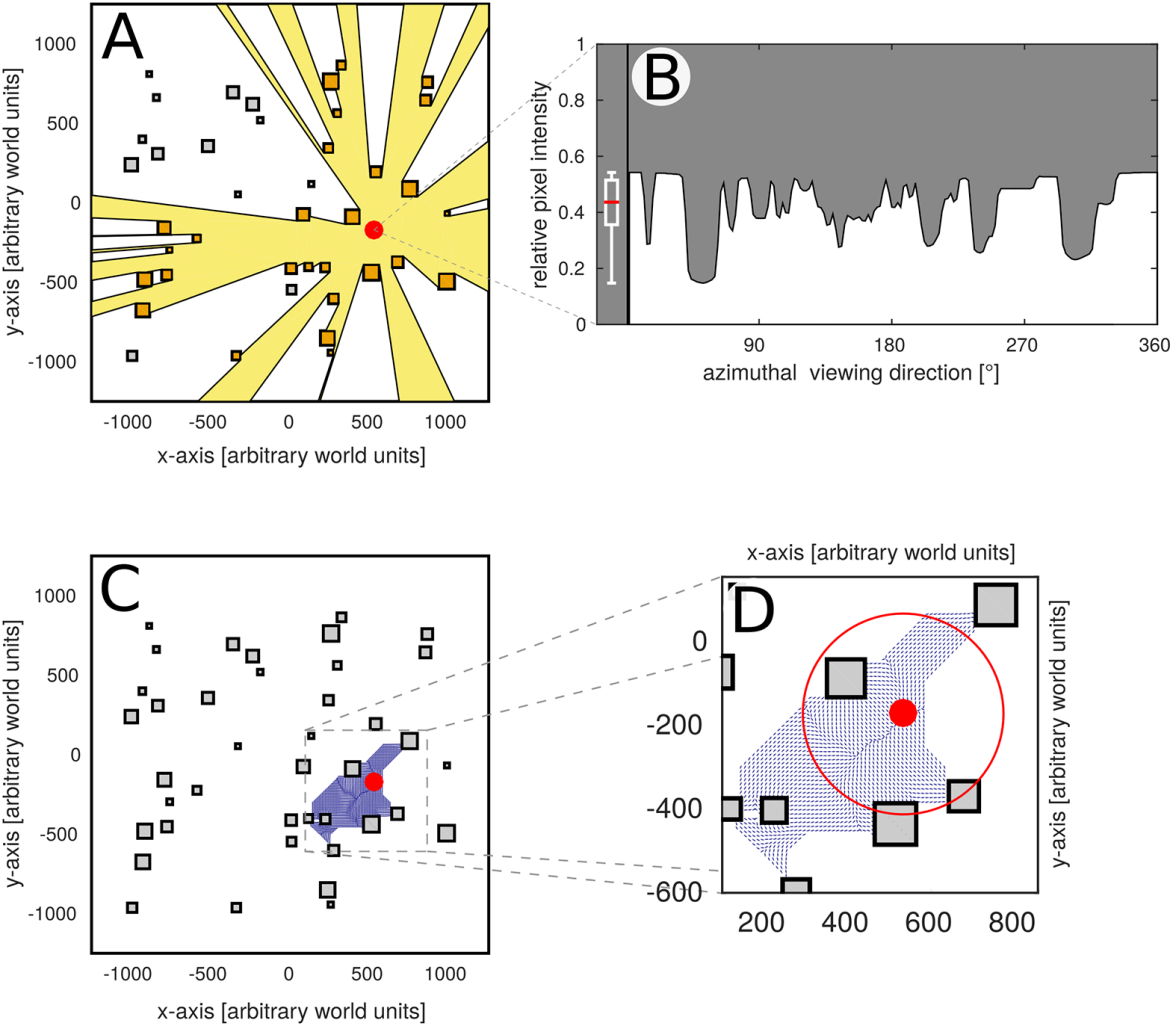
Methods overview. **A**: Top view of a simulated environment. Squares represent objects, the **yellow area** shows the isovist of the location marked by a **red dot**. The isovist contains the part of the environment from which a location is visible and thus quantifies the “visibility” of the homing target. Additionally, objects directly adjacent to the home location are highlighted in **orange**. The number and distance to adjacent objects (called “neighbours”) can be used to quantify the spatial clutter of a home location. **B**: Brightness skyline of the location highlighted in (**A**). Computing the median and interquartile range of the brightness of the skyline (box plot left of the skyline) can be used to quantify visual clutteredness of the home location. **C**: Same environment as **A**. When analysing the performance of a homing strategy, the area covered by all trajectories converging on a given home location is defined as its catchment area (**blue arrows**). The size of the catchment area can be used to quantify the reachability of a location. Here we take the catchment area and compute the radius of a circle with the same area. This results in the catchment radius of a given homing target, which gives an intuitive measure of reachability that is expressed by the same distance unit used for the world (see **Inset** to the right).

While all of the proposed models face and address different challenges, they have a common metric to assess their relative success in different environments. This metric is that of the catchment area [14] and describes that part of an environment, from within which homing will be successful. This means that if an animal tries to navigate to its nest from a given location in the environment and is able to reach it following a specific homing strategy, its starting location will be within the catchment area of its nest. Such catchment areas are often observed to differ in size for different home locations in an environment [6, 15, 23, 37], so that some locations can be reached much easier than others using a given strategy. For this reason, we will use the size of a home location’s catchment area as our metric of homing success (see Fig 2C).

We do have to note, however, that depending on the strategy used, larger catchment areas are often more shallow and thus more susceptible to noise-induced homing errors. Hence, there are potential trade-offs between catchment area size and the robustness of homing success [38, 39]. Nevertheless, for the homing strategy employed in this study, this issue can be neglected, as the way we compute homing directions excludes this type of noise-induced homing error (see Methods for a detailed description of the homing procedure).

Since homing success is expected to be related to different environmental characteristics [38, 40], we additionally quantify several different spatial and visual parameters of all home locations, to be able to assess whether different environmental parameters could be used to predict homing performance. Firstly, we quantify from which parts of the environment the home location will be visible for a navigating agent. The area covering all locations from which the home location is visible is called the isovist of that location [41] and has previously been linked to navigational behaviour in humans [42]. This allows us to determine systematically whether there is a link between the area from within which the goal is visible (isovist) and the area from which it can be reached (catchment area) (see Fig 2A vs 2C). In addition, we determine the number and distance to all objects directly adjacent to the home location (i.e. all objects which can be seen from the home location and which are not obstructed by other objects), which allows us to determine the degree of “clutteredness” of the home location (see Fig 2A, highlighted objects). Finally, we determine the variance and average brightness of the panorama as seen at the home location. This enables us to also quantify clutteredness visually in terms of image parameters. Using these characteristics, we will try to link the visual and spatial surroundings of the home location to its catchment area.

### Research questions

In summing up, we will address the following research questions:

1. How does homing performance (measured by the size of a home location’s catchment area) depend on the position of the home in the environment?
2. Can homing success be predicted by computationally inexpensive procedures that could plausibly be employed by a navigating animal?

## Results

### Simulation overview

To investigate the convergence properties of the ASV model and to address the question of home choice, we systematically analysed homing trajectories computed in a series of simulated cluttered environments. The environments consisted of 40 (for additional environments containing 80 and 120 objects see S3 Appendix) black cuboid objects ranging between 30 and 100 units in width and 100 to 800 units in height randomly placed on a black plane of 2500 by 2500 units width without overlapping (see Figs 1 and 2). The density of objects is comparable with the reconstructed ant habitat used by [23], so that the lateral length of 2500 units set for our environments could be interpreted as 2.5 meters in real world distance. Note, however, that this is an arbitrary interpretation and any other scaling would be equally valid.

This type of environment filled with numerous similarly looking objects, like the grass tussocks encountered by navigating ants, provides a challenging landscape for the homing algorithm. It should thus be well suited for our analysis of home choice.

Panoramic snapshots were taken along a 220 × 220 grid covering the entirety of the environment at a distance of 10 units between each snapshot (see Methods for a more detailed explanation of the image rendering process). These snapshots were processed according to the ASV model: every scene was encoded by a two-dimensional vector representing the spatial distribution of pixel intensity values across the panorama. By taking the difference between these vectors determined at both the home and the current location, a homing vector (*HV*) can be computed, which can be used to navigate through the environment (see Methods for a formal introduction to the ASV model). Iterating on this procedure, homing trajectories were determined from any grid position, traversing the grid to reach a given home location. All trajectories terminating at a given home location are considered to be in its catchment area. We then compute the radius of a circle with the same area (*catchment radius*) and use this as the measure of how well a given location can be reached (in the following called *reachability*). Note that this measure disregards the actual shape of the catchment area in favour of an “average” reachability from all directions.

To be able to link reachability to the structure of a home location’s surroundings, we determined its clutteredness both visually and spatially by quantifying different environmental parameters for each home location: The first of these is the area covering all parts of the environment from which the goal could be seen, also called the location’s isovist (see [41] for an introduction to the concept). Here a larger area means that the home location can be seen from a larger part of the environment. Furthermore, we determined the number of all objects that were direct neighbours of a given home location (i.e. directly visible from that location) and the distances to them. In addition to these spatial characteristics we also assessed the resulting visual clutter by analysing the average altitude of the brightness-skyline of the panorama as well as its variance taken at a given home location.

### Overall reachability

To assess the overall performance of the ASV algorithm in our cluttered virtual environments, a systematic analysis of catchment area sizes was performed for all home locations on the grid spanning the entirety of each simulated environment.

#### Average reachability is consistently low

**Fig 3:**
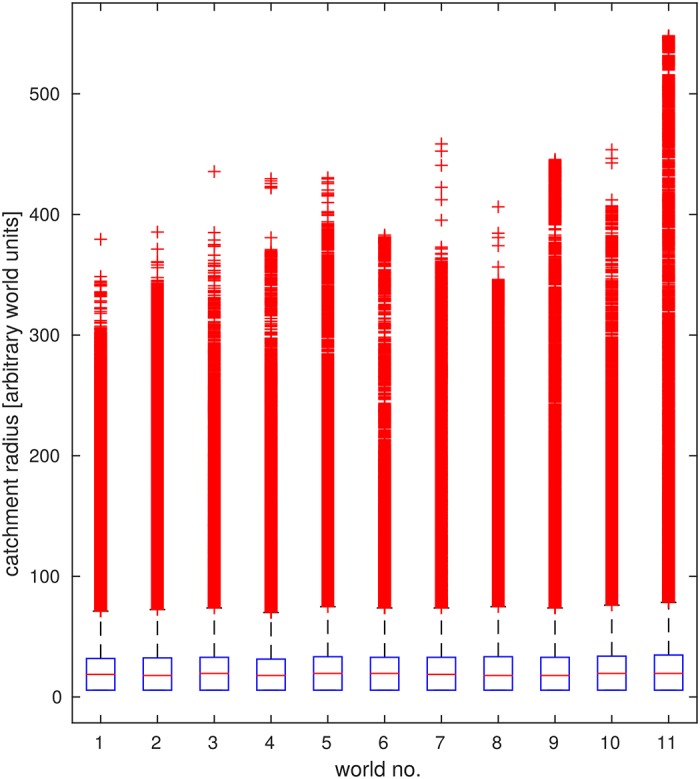
Overall homing success. Catchment radii quantify from which distance a location can be reached using a given homing strategy. Box plots show the distribution of catchment radii (in world units) for all target locations and 11 simulated environments analysed. The median catchment radius is outlined in **red**, outliers are shown as **red crosses**. Catchment radii were found to differ greatly for different locations in the environment; distributions are consistently heavy-tailed so that median catchment radii are consistently small at 17-20 units.

As can be seen in Fig 3, the median catchment radius is low at around 17-20 units in all tested environments. Furthermore, the distribution of catchment radii is heavily skewed towards small radii. However, a sizeable number of home locations exists which has a catchment radius of up to about 400 units (see Fig 3). The small median catchment radius indicates that, on average, successful homing is possible, but only in the vicinity of the home.

These results appear to be stable not only across all tested environments (see Fig 3), but also across different grid resolutions and for different tolerance levels that allow trajectories passing closely by the goal to be counted as converging (see S3 Appendix).

### Visuospatial analysis

In order to link the observed catchment radii to the visuospatial structure of a home location’s surroundings, different environmental parameters were quantified and related to the homing performance (see Methods for a more detailed description of the environmental parameters quantified and S2 Appendix for an overview of their relation to each other).

#### No clear link between visuospatial parameters and catchment area size

**Fig 4:**
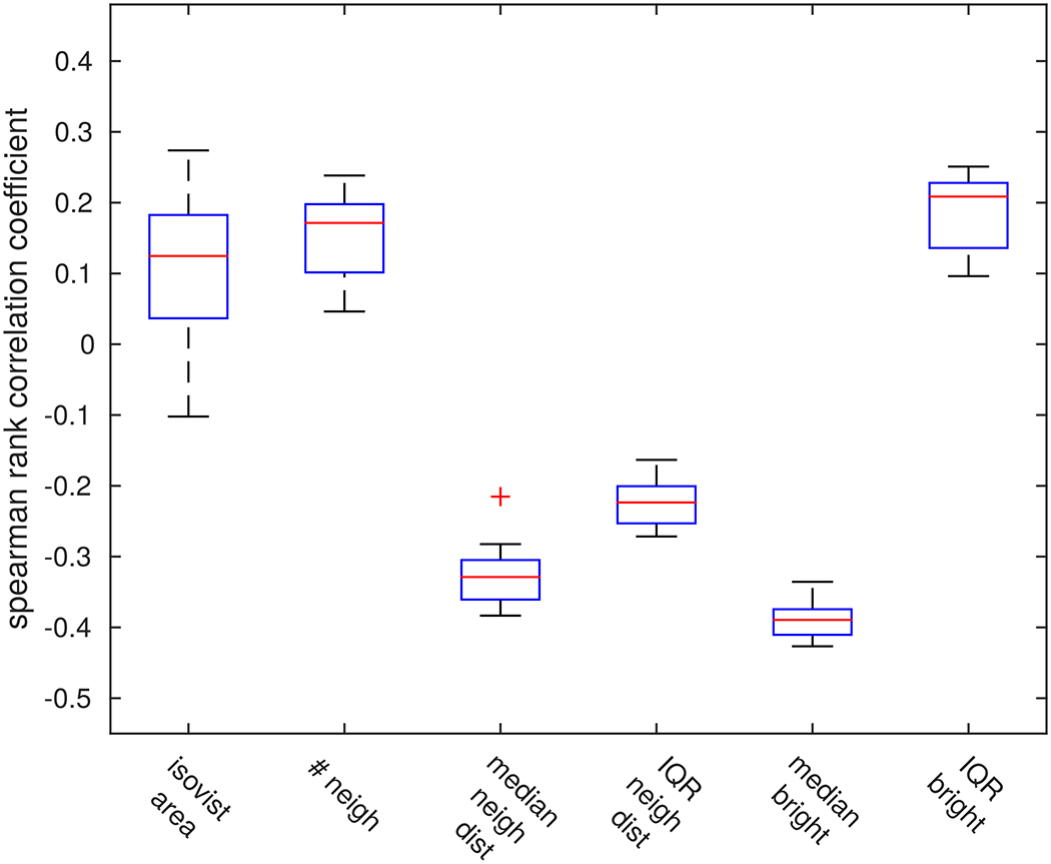
Relationship between environmental parameters and homing success. Box plots show the distribution of Spearman correlation coefficients between each of the computed environmental parameters and the catchment radius of the corresponding locations for all 11 analysed environments. None of the analysed parameters show a strong relation with the catchment radius.

Fig 4 shows the distribution of Spearman rank correlation coefficients between the respective environmental parameters and the catchment radii for each of the 11 environments tested.

The isovist area, that is the area from within which the home is visible, shows no clear relation with the catchment radius with a median rank correlation coefficient of 0.12 and a large divergence in correlation that may extend even into the negative range. The median correlation coefficient for the number of neighbours is similarly low at 0.17; however, here the range of observed correlation coefficients does not extend to negative values. This similarity also reflects the moderate positive correlation between the two parameters (median correlation coefficient 0.57, see S2 Appendix).

Furthermore, there appears to be a weak negative correlation between the median distance to a home location’s neighbours and the catchment radius (median correlation coefficient -0.33), but no clear relation between the catchment radius and the variance in neighbour distances (median correlation coefficient -0.22). The overall brightness of the scene—expressed as median skyline brightness—seems to be weakly negatively related to the catchment radius (median correlation coefficient -0.39), which matches the moderate correlation strength between skyline brightness and neighbour distance (median correlation coefficient of 0.58, see S2 Appendix). The variance of the skyline brightness profile does not seem to be clearly related to performance (median correlation coefficient -0.20).

Overall, none of the parameters quantified to describe the visual clutter of the environment show a strong relationship to the observed catchment radius. As such, none of them could readily be used to predict homing success.

### Local convergence pattern

As described above, the quantified visuospatial characteristics of a home location could not easily be related to homing success. One reason for this might be that to home successfully, the agent needs to iteratively approach its goal. Whether or not the agent will get lost on its way will be determined by the visuospatial characteristics all along its homing trajectory and not only by those at the goal. For this reason, we determined the local convergence pattern at the home location, to quantify how likely the agent would get lost on its way.

**Fig 5:**
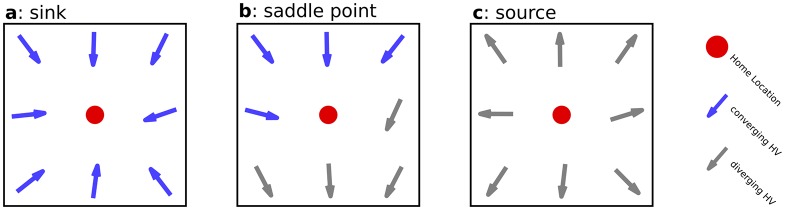
Home locations grouped by their local pattern of convergence. Homing vectors converging on the home location (*red dot*) are shown in *blue*, non-converging ones are shown in *grey*. Points reachable from all surrounding grid nodes are called sinks (**a**), those reachable from some, but not all directions are called saddle points (**b**). Points not reachable from any direction are called sources (**c**). We call these local patterns of convergence a location’s *target type*.

Due to the grid-based nature of our simulations, the directions of homing vectors close to the home location fall into one of three categories: A home location reachable from all directions is considered a *sink*, a location not reachable from any direction a *source* and a location reachable from some, but not all directions is classified as a *saddle point* (see Fig 5). We call this pattern of local convergence a home location’s *target type*. Based on theoretical considerations, we expect at least part of the home locations to be saddle points (see S1 Appendix). Thus, we determined the target type of all analysed home locations.

#### Local convergence is directionally limited for most home locations

**Fig 6:**
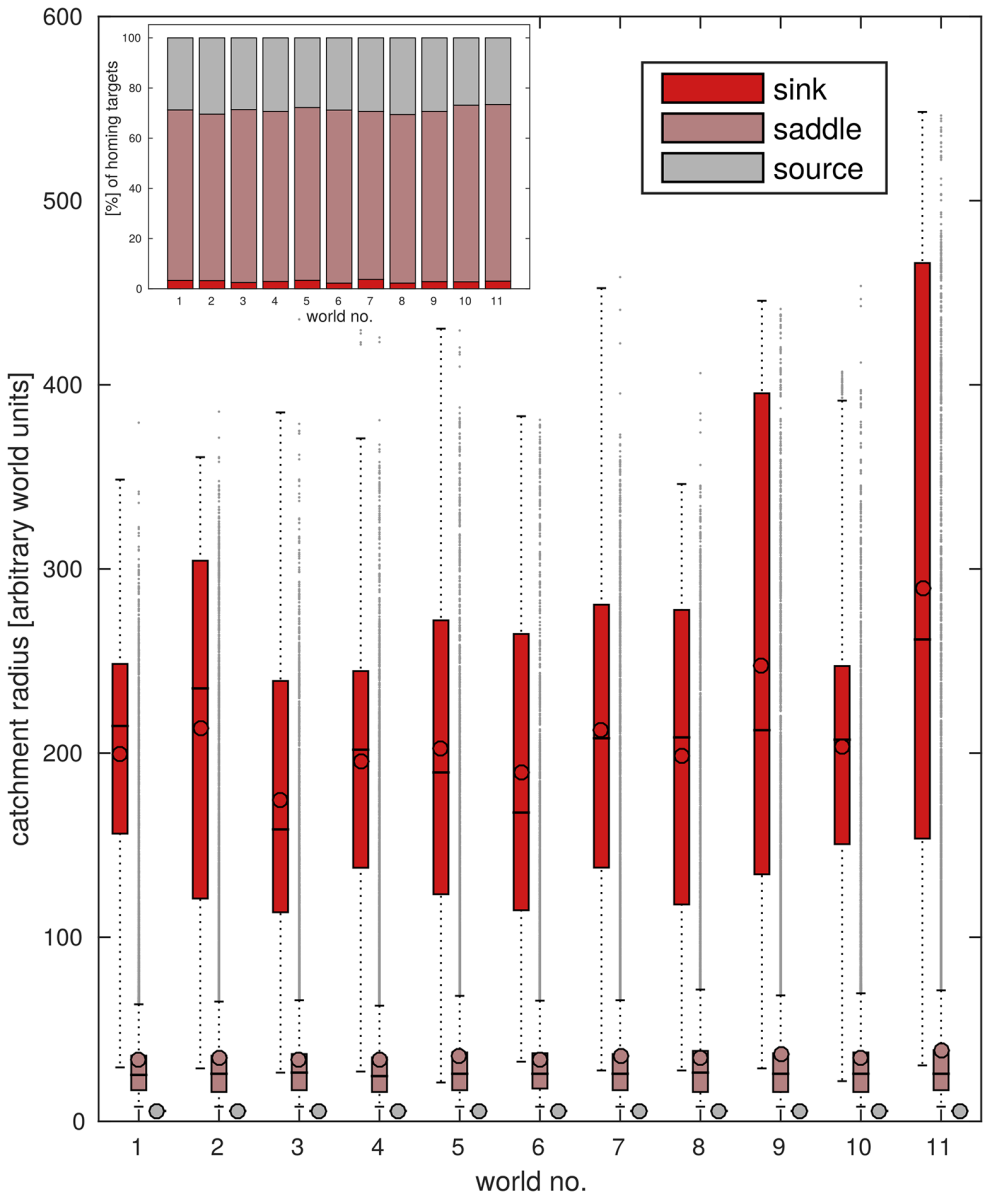
Reachability is greatly increased for sinks. Box plots show distributions of catchment radii for each target type (see also Fig 5) in each simulated environment. The inset in the top left shows the proportion of target types for the respective environments. The colour scheme shows sinks in deep red, saddle points in light red and sources in grey in both the inset and box plots **Inset**: Target type proportions are robust across environments, with saddle points making up about 70% and sources about 25% of home locations, leaving less than 5% of locations as sinks. **Box plots**: Distributions of catchment radii found for *saddle points* closely match those found overall, with a median of around 20 units and a long tail of outliers reaching up to around 500 units (cf. Fig 3). Catchment radii for *sources* are expectedly constant at ∼ 5 units. Strikingly, at around 200 units, median catchment radii for *sinks* are consistently an order of magnitude larger than the overall median catchment radii (cf. radii for saddle points) in all tested environments. Catchment radii are also more evenly distributed around the median. Results are robust across all tested environments.

The analysis of target types revealed that most home locations are saddle points (∼ 65-70%) and a smaller proportion are sources (∼ 25-30%), leaving less than 5% of locations as sinks (see Fig 6, Inset). We thus find no or only limited local convergence for more than 90% of all home locations. Furthermore, we find that this distribution of convergence patterns is consistent across all environments and for all tested step sizes (see S3 Appendix).

### Link between local convergence and catchment radius

When analysing the different target types individually, pronounced differences in the respective median and distribution of catchment radii become obvious. The median catchment radius for saddle points (∼ 25 units, see Fig 6, light red) is only slightly larger than the overall median (∼ 15-20 units, see Fig 3) and the distribution is also similar to that observed overall. This is expected, as saddle points make up roughly 70% of home locations. The catchment radius of sources is constant at ∼ 5 units, which is the radius of a catchment area covering only the home location itself (see Fig 6, grey).

#### Homing success for sinks is greatly increased

**Fig 7:**
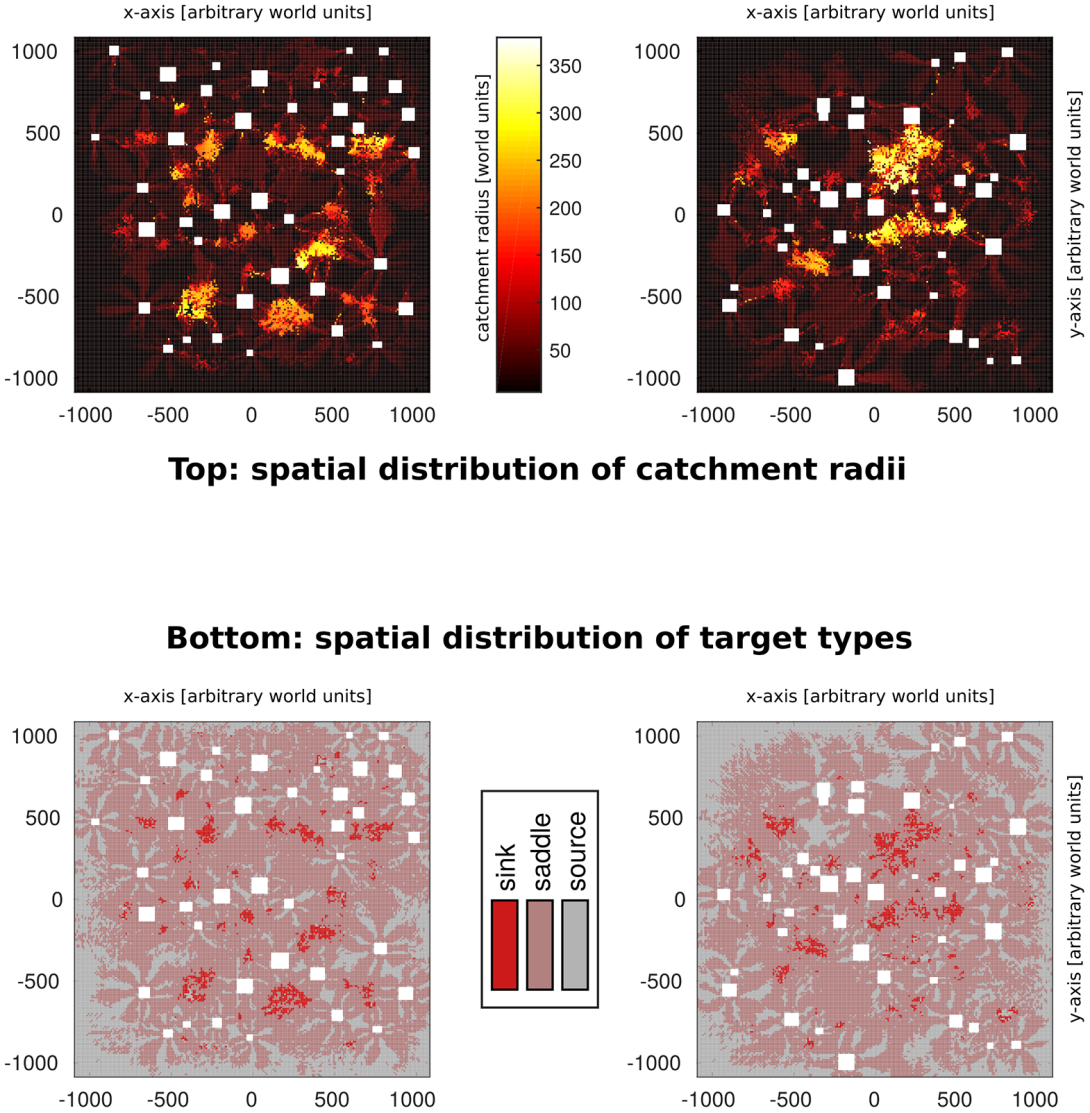
Spatial distributions of reachability and local convergence are similar. Top view of two of the simulated environments. Squares represent objects. **Top:** Catchment radii of locations are colour-coded, so that locations with larger catchment radii appear brighter than those with smaller ones. Overall, a grouping of larger catchment radii (*bright spots*) in clearings surrounded by constellations of multiple objects can be observed. There are however some locations characterised by large catchment radii outside these formations. Furthermore, locations very close to objects are generally characterised by very small catchment radii (*dark “halos” around objects*). **Bottom:** The local convergence pattern (target type) of each home location is shown in colour. Note the overlap between sinks and locations characterised by a large catchment radius (**Top**). These patterns are consistent across all tested environments.

In contrast, the median catchment radius of sinks amounts to around 200 units (Fig 6, deep red), making the average catchment radius of a sink almost 10 times larger than that of a saddle point. Moreover, catchment radii are much more evenly spread around the median for sinks. This marked difference in median catchment radius means that locally determining the target type of a home location allows to make sound predictions about its global reachability. Specifically, choosing a home which is a sink, will on average increase homing performance significantly. The link between larger-than-average catchment radii and the local convergence pattern is also visible in the overlap between the spatial distributions of sinks and home locations with large catchment radii (see Fig 7).

## Discussion

When using the ASV model, the reachability of a home location varies depending on its location in the environment. Hence, locations differ greatly in their quality as possible home sites. However, the environmental characteristics of a location cannot easily be used to predict the size of the catchment area and, thus, homing success. Nevertheless, it is of great interest to a navigating agent to be able to detect whether a location can be easily found or not. While the analysed visuospatial characteristics of the home location could not be used to predict homing success directly, the different local patterns of convergence (i.e. the target type) of a home location could be shown to be linked to the size of its catchment area. Most importantly, although locations that can be reached from all directions are relatively rare, they tend to have much larger catchment areas than the other target types. The local convergence pattern can thus be used to identify promising homing targets to increase homing success.

### Home choice

#### Overall homing success

Overall, most of the home locations analysed in our simulations were characterised by small catchment radii (see Fig 3), limiting their usefulness as homing targets. However, the existence of a subset of locations characterised by remarkably large catchment radii indicates that some locations in the environment will be singularly well suited as home locations to choose. It will thus be of great interest to a navigating animal, to identify these locations to improve its homing success.

#### Linking reachability and environmental structure

While the benefits of selecting a home location with a large catchment radius are readily apparent, it is implausible for an animal to probe its entire environment to assess a location’s full catchment area, as was done by the systematic analysis performed here. Are there ways for an animal to gain information on a location’s reachability without having to perform such an unrealistically exhaustive survey?

To investigate whether certain features of a home location’s environment might be used to predict homing success, we investigated the relationship between several spatial and visual parameters of a location’s surroundings and the reachability of that location. Firstly, we determined the area from within which a home could be seen, called its isovist [41, 42], since intuitively a location which is visible from a larger part of the environment might also be found more easily. Furthermore, we determined the distance to all directly adjacent objects for each home location (i.e. its “neighbours”). This allowed us to directly quantify the degree of spatial clutter for a given home. Lastly, the average brightness of the scene and the variance of the brightness skyline were computed in order to quantify the visual degree of clutter. However, when trying to relate these environmental parameters with a location’s reachability, no clear patterns emerged (see Fig 4). This means that none of the the derived parameters could be used to meaningfully predict catchment area size and, thus, homing success.

Nevertheless, if we consider the spatial distribution of locations with large catchment areas (see Fig 7), we observe a concentration of these home locations in clearings surrounded by objects at an intermediate distance. This indicates that there should be some relationship between the spatial structure of the environment and the observed homing success. However, as described above, deriving the topological characteristics which lead to this increased homing success tends to be very difficult. One reason might be, that to capture those characteristics of the environment which determine homing success, one needs to consider not only the home location itself, but also the route towards it.

#### Identifying promising home locations

When determining the directions from which a given home location could be reached using the employed homing algorithm, we discovered that the local pattern of convergence is a useful predictor of the overall reachability: Sinks, on average, possess considerably larger catchment radii than saddle points (median catchment radius is increased by an order of magnitude, see Fig 6). This implies, that a navigating agent can select a sink as home to improve homing success.

Hence, if an animal determines the local convergence pattern of a given location, it might use this information to predict the catchment radius and, thus, the quality and usefulness of the location as a home site. If the location can be reached equally well from all directions, it is likely a promising home location to memorise.

From a behavioural point of view, the local convergence pattern of a potential home location could be determined by a chain of “homing trials” performed in its immediate vicinity. At various trial points in the vicinity of the home location, the homing direction could be computed and compared with the direction in which the actual home location is located. If the directions match, the trial would be successful and homing from this direction would be possible. If trials are successful all around the home location, it can be reached from all directions and will most likely be a good location to select. Interestingly, bees and wasps perform a behaviour compatible with this idea when first leaving the nest or encountering a new food source [43–46]. This behaviour, often called a learning flight, has been associated with the acquisition of information about the local environment [47, 48], and even though in these cases the location of the home has already been fixed, this illustrates that bees do possess a behavioural repertoire, which could also be used to perform “homing trials” to assess a location’s local pattern of convergence.

Furthermore, since individual saddle points may also be characterised by large catchment radii (Fig 6) and since saddle points make up the majority of all home locations (Fig 6, inset), it might also be of interest to investigate whether ants or other central place foragers preferentially approach such locations from those more reachable directions determined by the local convergence pattern. It is already known that ants can make use of the wind directions to extract compass information [49] and to inform their search behaviour [50], resulting in an directionally constrained homing and search behaviour. It might thus be interesting to investigate whether visually driven approaches might also be performed in a directionally selective way that conforms to the reachability of a homing target.

### Limitations of local homing strategies

Due to their reliance on comparing the current vista with that memorised at the homing target, local homing strategies are inherently limited in the range over which they can provide sufficient guidance. Thus, to be able to navigate over longer distances, an animal would have to recognise not only its final goal, but also intermediate locations en route (see for example [51] for a discussion of this topic). A possible way of implementing route navigation by following a chain of “target locations” set as way-points has been described and successfully tested by Smith et al. [52]. Their method of linked local navigation allows an agent to home towards a series of locations along a memorised route, switching from one way-point to the next. When using such a strategy, we might expect a navigating animal to select way-points in a way that minimises the number of locations which need to be memorised. Thus, the reachability of selected way-points will be of great importance, and we can expect animals to preferentially pick way-points with a large catchment radius, so that fewer intermediate locations have to be remembered. A promising way to investigate this hypothesis might be to compare the average catchment radius of on-route vs. off-route locations, for instance, of ant homing trajectories. Indeed, reconstructed routes of ants in a cluttered environment (see Figure 1 in [23]) with a similar density of objects as used in our analysis led through many surrounded places and, thus, might contain several locations characterised by large catchment radii (cf. bright spots in Fig 7). Then, if ants indeed use such a method of following linked way-points along a route, their course through the cluttered environment could be explained in terms of optimising way-point reachability.

Considering our results, one way to minimise the number of way-points required, might be to assess the local convergence pattern of possible way-points and preferentially select sinks as intermediate homing targets. Since the choice of a way-point is subject to fewer ecological constraints than the choice of a nest site, the animal is also likely to be more flexible in its choice. Mangan [25] reports a high degree of variance in the size of catchment areas observed along a reconstructed ant route when using a similar model to the one employed in this study. We might speculate, that an analysis of the target type of the locations chosen along the reconstructed ant path might be used to explain this observed difference in homing success for different parts of the route. In this context, an analysis of the way-points used by actual ants would be of great interest to further investigate the importance of target types for local homing procedures.

One alternative to way-point-based route following methods is the visual compass-like scene familiarity model described by Baddeley et al. [18]. Here, an artificial neural network is trained on all views experienced along an initial return trajectory. On subsequent trips the agent is then able to assess whether or not it is on or near its route, using the similarity between its current view and the holistic memory of its previous trajectory. This means that homing will be successful within a visual corridor around its previous path, the width of which is defined by whether the familiarity algorithm will lead the agent to converge on its route or not. Considering this, it would be interesting to investigate the relationship between reachability and target types of locations within such a visual corridor and its spatial extent.

### The ASV model

#### Grid-based homing

When aiming to systematically address navigation across large environments, a grid-based simulation approach is computationally most sensible and has been the method of choice for a variety of different modelling studies (e.g. [23–25, 38, 39, 53]). That is because instead of having to render hundreds or thousands of images for each individual trajectory, a limited set of panoramas can be pre-rendered and the derived image features saved to be used for all homing trajectories traversing the given location. This drastically reduces computation time and allows for a more systematic analysis of homing trajectories than would otherwise be possible.

However, from a biological perspective this approach is of course quite unrealistic. While a navigating animal might follow a strategy of repeated bouts of forward movement, interspersed with periods of reorientation, it is highly unlikely to observe a truly grid-like pattern of exactly spaced intervals under natural conditions. Furthermore, the discretisation imposed by the grid also distorts trajectories to some extent, as the simulated animals do not move exactly in the direction indicated by the homing vector of a given position, but rather to the neighbouring grid node most closely aligned with it. These distortions will also depend on the resolution of the grid. To illustrate this, one might imagine a perfectly continuous solution. Here, the agent will always move exactly according to the homing vector determined for its current location. This solution is equivalent to a grid-based solution of infinite resolution. Then, as the grid becomes coarser, more and more “nodes” on the original grid are lost. However, since each of these nodes might have sent the animal in a different direction, the trajectory taken by the animal across the same part of the environment will likely change as grid nodes are lost. Thus, to avoid possible biases induced by investigating homing only for a specific grid resolution, trajectories were computed systematically for a wide range of different grid resolutions. Our findings suggest a robust distribution of target types and catchment radii across a wide range of different parameters, while individual trajectories might change significantly for different grid resolutions (see step size sketch in S3 Appendix). This suggests that the choice of grid resolution is unimportant when performing a large-scale analysis as was done in this study. However when analysing individual trajectories, the chosen grid resolution might markedly impact homing success and the effect of this should be considered for the interpretation of any obtained simulation results.

#### Limitations of convergence

According to the theorem presented in S1 Appendix, convergence on all home locations is directionally limited. However, when analysing the distribution of target types, we observe a small but consistent proportion of sinks, i.e. home locations to which successful homing from all directions is possible. To explain this, we need to consider the details of the landmark vector computation. In the ASV model, “landmark features” are derived by extracting the average pixel intensity along the columns of the image. Thus, “landmarks” do not directly correspond to specific, spatial features in the environment, but associate an intensity value with a given direction. Hence, there are no fixed “landmarks” detected at certain positions in the environment, as it is assumed by the theorem. Furthermore, the discrete nature of our simulations deviates from the continuous case assumed by the theorem. Thus, there is a discrepancy between the observed convergence patterns and the prediction derived from the theorem. Still, over 90% of all locations are not reachable from all directions, with more than 70% of these locations being saddle points (see S3 Appendix). Hence, the presented theorem still aids in the understanding of observed patterns of convergence and constitutes a valuable contribution to the understanding of the presented homing method.

#### Feature-dependence of performance

In our simulations, pixel intensity values were used for the computation of the ASV to encode the visual panorama at a given location. However, the principle of vectorial summation derived from the original ALV model is not limited to this specific type of feature.

Firstly, the panoramic view can be encoded in different ways other than through vertical averaging of pixel intensities. For example, as described by [26], the skyline might be extracted directly, by segregating the input image via different colour channels. The principle might even be applied without any step to extract a skyline, utilising the full image to directly compute an ASV that also takes into account the elevation (then more appropriately called an average scene rather than skyline vector). This would allow for 3-dimensional homing, similar to the procedure described by Murray & Zeil [39] for catchment volumes of image difference functions. Furthermore, other visual cues, such as optic flow-based nearness estimates, could be used in a similar manner, substituting pixel intensity with flow amplitude values to determine a “nearness panorama” [54].

Secondly, the same principle may also be applied in non-visual contexts. All that is required for the method to work, is for the extracted feature to be translated into a magnitude value associated with a given direction. Thus, if for example auditory or olfactory information is available in a direction-selective manner (see for example [55]), it might also be used for navigational purposes with the ASV model.

However, different extracted features (especially the ones associated with different sensory modalities) likely differ in their spatial distribution. Thus, we might expect different levels of reachability for a given location, depending on the feature used. This means that, for example, a nest entrance marked by an arrangement of “landmarks” might be easily reachable using visual information, however its profile as determined by a different cue (like olfaction) might be less unique, impeding successful homing. Thus, if multiple cues could be used for navigation, “homing trials” might also serve to ascertain which of the available cues will be most useful for homing to an already established home. This could be considered another reason for performing learning flights in the vicinity of a nest or food source as observed for example in bees (see above). In this context, the combination of homing vectors derived from different sensory cues to improve homing performance might be a topic of interest for further study.

## Conclusion

We could show that the question of home choice is likely to be an important factor in the context of navigation and an interesting problem worthy of further investigation. Particularly, different ways a navigating animal might assess a location’s quality as a home should be explored. While we could show that ensuring omni-directional local convergence might be one such way, it is likely that a variety of different features play a role for this kind of assessment. Exploring such different features (like the idea of surroundedness mentioned above), will surely yield valuable insights into the way different types of spatial information may be used by navigating animals.

Furthermore, while the obtained results highlight the usefulness of simulation studies for systematic analyses, the comparison of theoretically predicted and behaviourally obtained catchment areas is necessary to further our understanding of animal navigation strategies. Thus, the study of nest placement from a navigational point of view should be pursued further by investigating the real-world catchment areas of nests and other homing targets used by different animals such as ants, bees or even birds.

## Methods

Simulations and analyses were performed using the Matlab^®^ software environment.

### Simulation and image rendering

To systematically test the ASV model, a series of 11 virtual 3D-environments was created. The environments consisted of 40 cuboid objects ranging between 30 and 100 units in width and 100 to 800 units in height randomly placed on a plane of 2500 by 2500 units width without overlapping. Panoramic images were computed using the CyberInsect toolbox [36] at each location of a grid with 220 × 220 equidistant locations spread across the entire environment with a spacing of 10 units. This led to a total of 48400 panoramic images.

At each location, the computed image covered 360° in the horizontal and 180° in the vertical (-90° to 90°, with 0° at the horizon) plane with a resolution of 2° in both dimensions, resulting in 180 × 90 pixel panoramas (Fig 1). Panoramas were 8-bit grey-scale images, with a resolution of 2° that reflects the spatial resolution of insect visual systems (e.g. [56, 57]), while at the same time reducing processing demands for possible hardware implementations. Furthermore, the grid-based approach allowed for a high spatial resolution in covering the entirety of the environment while keeping computational demands manageable, since it made it possible to pre-compute and store panoramic images all across the environment for later use without having to re-render the environment for every homing trajectory.

#### Average Skyline Vector computation

To compute the Average Skyline Vector (ASV) of a given scene, the pixel intensity (or brightness) of each image column was summed along the elevation, yielding an average brightness value for each azimuthal direction. Since our environment was covered entirely with black-and-white textures, this vertical summation of pixel intensities was equivalent to an explicit extraction of the skyline altitude and thus combines the “elevation” and “skyline” codes described in [23]. Taking these average brightness values as the norm and the viewing direction as the argument results in a list of skyline vectors (SVs) encoding the whole panorama:

$$\begin{array}{ccc} ∥\vec{SV}\left(Az,p\right)∥ & = & \sum_{El}I\left(Az,El,p\right)\\ \arg\left(\vec{SV}\left(Az,p\right)\right) & = & Az \end{array}$$ (1)

where *I* is the observed pixel intensity in the viewing direction determined by the horizontal (*Az* = {0, …, 360}) and vertical (*El* = {−90, …, 90}) viewing angle at location *p*. Summing these skyline vectors yields the average skyline vector (ASV):

$$\begin{array}{c} \vec{ASV}\left(p\right)=\sum_{Az}\vec{SV}\left(Az,p\right). \end{array}$$ (2)

Since the norm of each skyline vector is determined by the average brightness value corresponding to a given viewing direction, it changes depending on the distance to objects. That is, as an object is approached it will subtend a larger visual angle on the retina in the given viewing directions, thus reducing the length of the respective SVs. This is an important feature of this type of skyline-based model, as it should negatively impact convergence of the homing algorithm (see S1 Appendix).

One thing that has to be noted, is the fact that all images processed in this way have to be aligned beforehand. While this is given in our simulation, a biological agent would first have to determine the correct alignment via mental or physical rotation of a given scene. To explain how an animal might solve this problem, visual compass methods have been described [29, 30, 38] that might allow it to perform the necessary alignments.

#### Computation of homing trajectories

Homing vectors (HVs) were computed by subtracting the ASV of the home location from the one determined at the current location:

$$\begin{array}{c} \vec{HV}\left(p\right)={\vec{ASV}}_{p}-{\vec{ASV}}_{p^{\prime }}, \end{array}$$ (3)

with *p* being the current location and *p*′ the home location.

This means, that for any given home location, the HV for all other locations on the grid could be computed. These HVs could then be used to generate grid-based trajectories simulating navigation through the environment. To this end the agent was moved along the grid, starting from a given location and moving to the neighbouring location most closely aligned with the homing direction stored in the HV computed for its current location. This reflects the procedure described by previous studies using the same algorithm [23–25]. However, while the mentioned studies focused on a small number of home locations placed along reconstructed routes, we were interested in trajectories covering the whole environment to perform a more systematic analysis. Hence, not only selected individual locations, but all 48400 locations on the grid of all 11 environments were used as home locations, and homing trajectories starting from all 48400 locations were computed for each of them. This resulted in a set of 48400^2^ trajectories for each environment.

### Assessment of environmental parameters

In order to potentially link the observed homing success of a given location to the visual and spatial structure of its surroundings, we determined several environmental parameters. This allowed us to determine the degree of visual and spatial clutter for each location. To this end we computed the area covered by that part of the environment from which the home location would be visible, also called the location’s isovist [41, 42], using the *Visilibity* toolbox [58]. A less cluttered location will potentially have a greater isovist, meaning it can be seen from a larger part of the environment. We also determined the number and distances to all objects directly adjacent (i.e. within direct line of sight) to the home location, thus quantifying spatial clutter more directly. Furthermore, to assess clutter visually, the average brightness and azimuthal variance in brightness of the panorama taken at the respective home location was calculated.

These measures where then correlated with each other and with the respective catchment radius to determine, whether the visuospatial make-up of a home location’s surroundings could be used to predict homing success.

### Data analysis

#### Local convergence pattern

Theory predicts that convergence on home locations will be limited to certain directions, as home locations may be saddle points (see S1 Appendix). To investigate the prevalence of saddle points, home locations were categorised by their convergence pattern (see Fig 5): A grid position was considered either a sink (i.e. all 8 neighbouring grid positions converge on the home location), a source (none of the 8 neighbouring grid locations converge), or a saddle point (some, but not all neighbouring grid locations convergence). We call this the *target type* of a given home location.

#### Reachability

In accordance with [23], the area covered by all trajectories converging on a given home location was called the catchment area of that location. We then computed the radius of a circle with the same area as the catchment area to derive a catchment radius of that location. Note that this disregards the actual shape of the catchment area in favour of an “average” reachability from all sides (see also Fig 2).

## Supporting information

S1 Appendix Proof of limited convergence. The Appendix contains a formal proof of limited convergence for ALV variants using distance-dependent weights.
(PDF)

S2 Appendix Statistical analysis of environmental parameters. This supplement contains results for statistical tests determining the relation between the different environmental parameters.
(PDF)

S3 Appendix Results for additional simulations. The supplementary results contain an analysis of additional simulations determining the effect of changing different simulation parameters.
(PDF)
