# Supplementary material for: The problem of home choice in skyline-based homing
Source: PLoS One. 2018 Mar 9;13(3):e0194070. doi: 10.1371/journal.pone.0194070 (PMC5844572; doi:10.1371/journal.pone.0194070)
Supplement: S1 Appendix — The Appendix contains a formal proof of limited convergence for ALV variants using distance-dependent weights. (PDF) [file pone.0194070.s001.pdf]

S1 Appendix: Proof of limited convergence

We assume that the agent's local coordinate system is aligned with the world coordinate system. Then let $\mathbf{x} = (x, y)^T$ be the agent's current location, and $\mathbf{x_0} = (x_0, y_0)^T$ the agent's home location. For every landmark $i = 1, \ldots, n$ detected by the agent at location $\mathbf{x_i} = (x_i, y_i)^T$, a *landmark vector* pointing from the agent's location to the landmark can be defined as $\mathbf{L_i}(\mathbf{x}) = \frac{\mathbf{x_i}-\mathbf{x}}{\|\mathbf{x_i}-\mathbf{x}\|}$.

The original ALV model as stated by [1] computes the home vector as

$$H(\mathbf{x}) = \sum_{i=1}^{n} \left(\mathbf{L_i}(\mathbf{x_0}) - \mathbf{L_i}(\mathbf{x})\right). \tag{1}$$

Here we use additional weighting terms w_i and w_i^0 to model the influence of each landmark on the home vector computation.

$$H(\mathbf{x}) = \sum_{i=1}^{n} \left(w_i^0 \mathbf{L_i}(\mathbf{x_0}) - w_i \mathbf{L_i}(\mathbf{x})\right). \tag{2}$$

For unweighted landmarks, i.e. $w_i = 1$ for all i, it can be shown that trajectories following the home vector field $H(\mathbf{x})$ converge on the home location $\mathbf{x_0}$ [2].

Using a distance-variant weight for each landmark — for example the height h_i — the weight w_i at the home location $\mathbf{x_0}$ and the current location $\mathbf{x}$ can be chosen as $w_i^0 := \frac{h_i}{\|\mathbf{x_i}-\mathbf{x_0}\|}$ and $w_i := \frac{h_i}{\|\mathbf{x_i}-\mathbf{x}\|}$.

Substituting these weights into $H(\mathbf{x})$, we obtain

$$H(\mathbf{x}) = \sum_{i=1}^{n} h_i \left(\frac{\mathbf{x_i}-\mathbf{x_0}}{\|\mathbf{x_i}-\mathbf{x_0}\|^2} - \frac{\mathbf{x_i}-\mathbf{x}}{\|\mathbf{x_i}-\mathbf{x}\|^2}\right). \tag{3}$$

As shown by the following theorem, all stationary points of $H(\mathbf{x})$ are saddle points. Since $H(\mathbf{x_0}) = 0$, the home location is a stationary point and therefore a saddle point.

Theorem 1. *Every stationary point in the home vector field $H(\mathbf{x})$ is a saddle point.*

Proof. The function $U(\mathbf{x})$, which fulfils $\nabla U(\mathbf{x}) = -H(x)$, is called the potential of $H(\mathbf{x})$. To determine sources, saddle points, and sinks in the vector field we can equivalently search for maxima, saddle points, and minima, in its corresponding

potential. For $H(\mathbf{x})$, the potential is given by

$$U(\mathbf{x}) = -\sum_{i=1}^{n} h_i \left(\ln(\|\mathbf{x_i} - \mathbf{x}\|) + \mathbf{x}^T \frac{\mathbf{x_i} - \mathbf{x_0}}{\|\mathbf{x_i} - \mathbf{x_0}\|^2} \right). \tag{4}$$

To compute the Hessian matrix $M(\mathbf{x}) = \begin{pmatrix} \frac{\partial^2}{\partial x^2} U(\mathbf{x}) & \frac{\partial^2}{\partial x \partial y} U(\mathbf{x}) \\ \frac{\partial^2}{\partial x \partial y} U(\mathbf{x}) & \frac{\partial^2}{\partial y^2} U(\mathbf{x}) \end{pmatrix}$ of $U(\mathbf{x})$, we first have to compute the second derivatives:

$$\frac{\partial^2}{\partial x^2} U(\mathbf{x}) = \sum_{i=1}^{n} h_i \frac{(x_i - x)^2 - (y_i - y)^2}{\|\mathbf{x_i} - \mathbf{x}\|^4} \tag{5}$$

$$\frac{\partial^2}{\partial y^2} U(\mathbf{x}) = \sum_{i=1}^{n} h_i \frac{(y_i - y)^2 - (x_i - x)^2}{\|\mathbf{x_i} - \mathbf{x}\|^4} \tag{6}$$

$$\frac{\partial^2}{\partial x \partial y} U(\mathbf{x}) = 2 \sum_{i=1}^{n} h_i \frac{(x_i - x)(y_i - y)}{\|\mathbf{x_i} - \mathbf{x}\|^4} \tag{7}$$

Since we have $\frac{\partial^2}{\partial x^2} U(\mathbf{x}) = -\frac{\partial^2}{\partial y^2} U(\mathbf{x})$ and $\frac{\partial^2}{\partial x \partial y} U(\mathbf{x}) = \frac{\partial^2}{\partial x \partial y} U(\mathbf{x})$, the second order principal of $M(\mathbf{x})$ is given by

$$\det(M(\mathbf{x})) = \frac{\partial^2}{\partial x^2} U(\mathbf{x}) \frac{\partial^2}{\partial y^2} U(\mathbf{x}) - \frac{\partial^2}{\partial x \partial y} U(\mathbf{x}) \frac{\partial^2}{\partial x \partial y} U(\mathbf{x}) \tag{8}$$

$$= -\left(\frac{\partial^2}{\partial x^2} U(\mathbf{x})^2 + \frac{\partial^2}{\partial x \partial y} U(\mathbf{x})^2 \right) \leq 0. \tag{9}$$

For $\det(M(\mathbf{x})) < 0$, the Hessian matrix $M(\mathbf{x})$ is indefinite and therefore $\mathbf{x}$ a saddle point. For $\det(M(\mathbf{x})) = 0$, it follows that $\frac{\partial^2}{\partial x^2} U(\mathbf{x})^2 = \frac{\partial^2}{\partial x \partial y} U(\mathbf{x})^2 = 0$, from which we obtain $M(\mathbf{x}) = 0$. In this case $\mathbf{x}$ is a flat point, which is a special case of a saddle point.

□

Divergence from simulation approach

As described above, extracting features to use as landmarks is a non-trivial task, which the ASV model avoids by directly using a low-level image statistic, in our case the average pixel intensity, computed from every image column. This process is appealing from a biological perspective as it corresponds to the extraction of a skyline, which is thought to be a process used by many insects for navigational purposes. Hence, in our simulation, there are no fixed landmarks but rather each image column is defined as a

single landmark. We thus write

$$\mathbf{x_i} = w_i \begin{pmatrix} cos(i\Delta\phi) \\ sin(i\Delta\phi) \end{pmatrix} \tag{10}$$

with $\Delta\phi$ being the angular resolution of the panoramic view. We therefore observe some mismatch between the presented proof and our simulation results.

References

1. Lambrinos D, Möller R, Labhart T, Pfeifer R, Wehner R. A mobile robot employing insect strategies for navigation. Robotics and Autonomous systems. 2000;30(1):39–64.
2. Möller R. Insect visual homing strategies in a robot with analog processing. Biological Cybernetics. 2000;83(3):231–243.
