# Supplementary material for: The problem of home choice in skyline-based homing
Source: PLoS One. 2018 Mar 9;13(3):e0194070. doi: 10.1371/journal.pone.0194070 (PMC5844572; doi:10.1371/journal.pone.0194070)
Supplement: S2 Appendix — This supplement contains results for statistical tests determining the relation between the different environmental parameters. (PDF) [file pone.0194070.s002.pdf]

S2 Appendix: Analysis of environmental parameters

Parameter overview

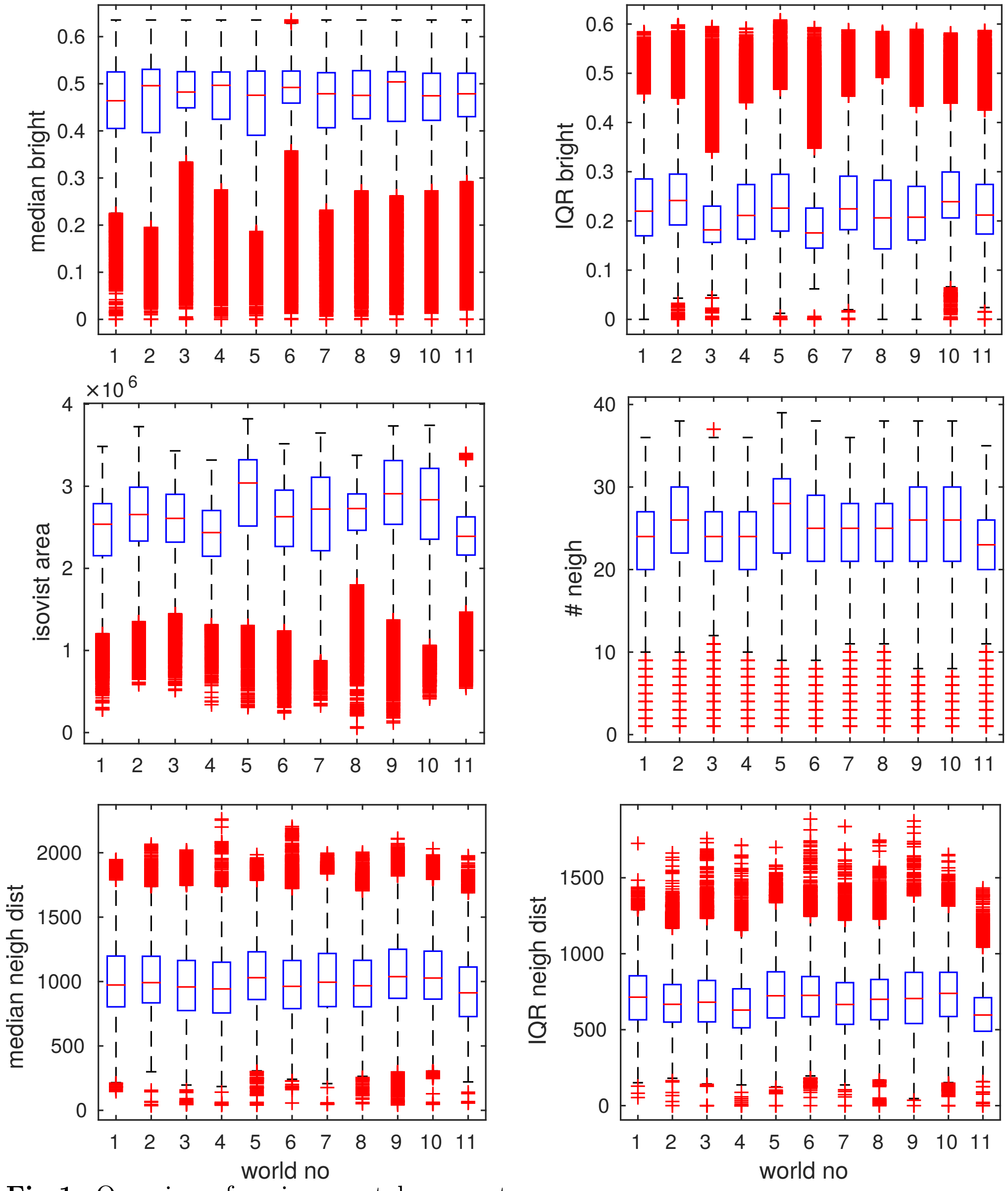

Fig 1. Overview of environmental parameters

Box plots represent the distribution of several different spatial and visual parameters computed for all home locations (n=48400) in each world (n=11).

We determined the variance and average brightness (between 0 and 1) of the panorama taken at the home location. This enables us to quantify' visual clutter in terms of image parameters (*median bright*, *IQR bright*).

We also quantified from which parts of the environment the home location will be visible for a navigating agent. The area covering all locations from which the home is visible is called the isovist of that location (*isovist area* in world units).

In addition, we determine the number and distance to all objects directly adjacent to the home, which allows us to determine the degree of spatial clutter of the home location (*# neigh*, *median neigh dist*, *IQR neigh dist*, IQR = interquartile range). The box plots above show that distributions are similar for all parameters, with isovist area being the most variable (see also parameter ratios below).

Parameter ratios

In order to compare the different environmental parameters across the different environments, the pairwise ratios between the medians of each parameter was computed for all worlds. The distributions of these ratios can be seen below. None of the ratios was found to be significantly different from 1 (Wilcoxon signed rank test, all results N.S., see below).

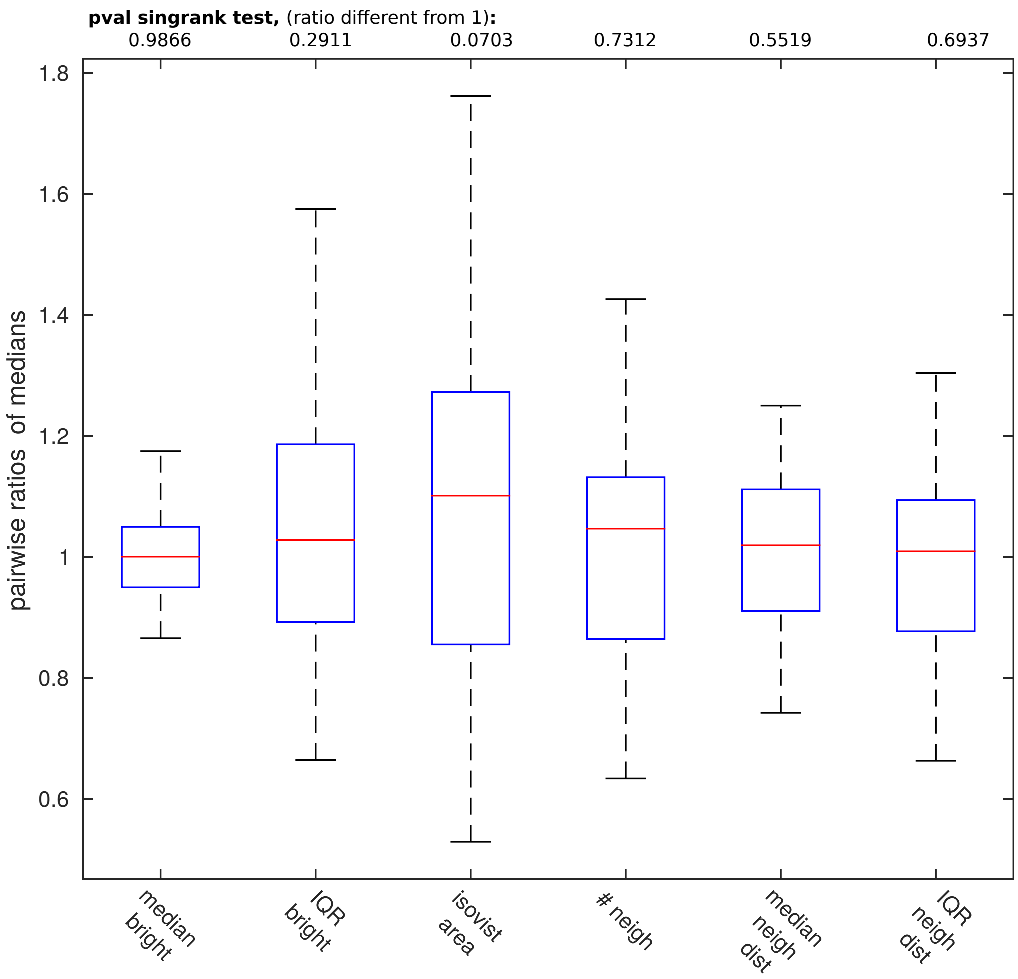

Fig 2. Pairwise ratios of parameter medians across all worlds.

Parameter correlations

Furthermore, the correlations between the different environmental parameters we determined. The box-plots below show the distributions of Spearman rank correlation coefficients between all parameters for all worlds (i.e. each box-plot shows the distribution of correlation coefficients observed between the two parameters in question for all worlds). Note that all possible combinations are shown, so that the upper and lower triangle of all presented box-plots show identical data.

We find moderate correlations between isovist area and number of neighbours (median correlation coefficient 0.57), as well as median neighbour distance and median skyline brightness (median spearman correlation coefficient of 0.58).

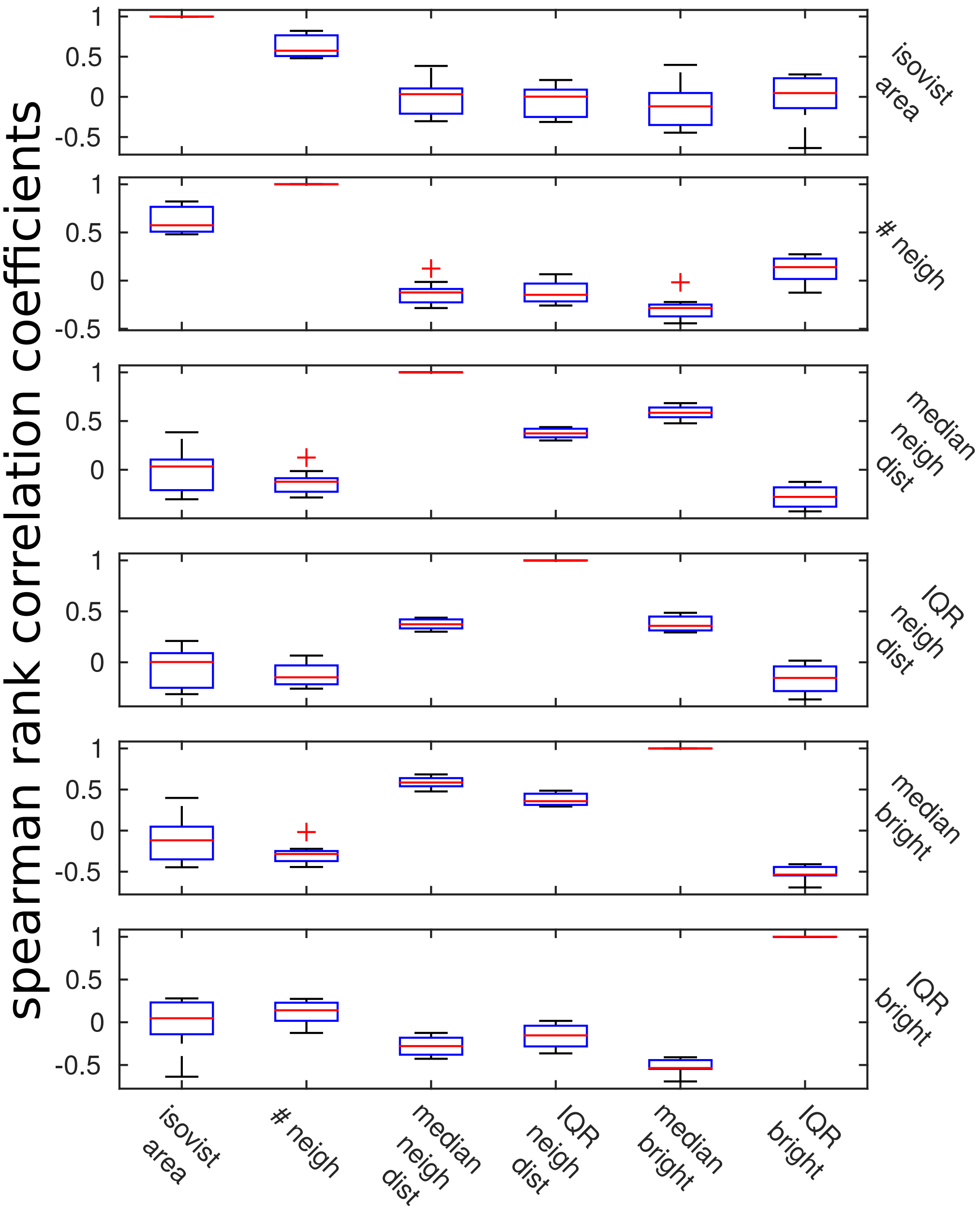

Fig 3. Pairwise rank correlation coefficients across all parameters and worlds.
