# Supplementary material for: The problem of home choice in skyline-based homing
Source: PLoS One. 2018 Mar 9;13(3):e0194070. doi: 10.1371/journal.pone.0194070 (PMC5844572; doi:10.1371/journal.pone.0194070)
Supplement: S3 Appendix — The supplementary results contain an analysis of additional simulations determining the effect of changing different simulation parameters. (PDF) [file pone.0194070.s003.pdf]

S3 Appendix: Additional Results

A grid-based simulation approach is computationally most sensible when aiming to systematically address navigation across a large environment as it allows a more efficient computation of homing trajectories (see for example [1–6]). However, from a biological perspective this approach is quite unrealistic. While a navigating animal might follow a strategy of repeated bouts of forward movement, interspersed with periods of reorientation, it is highly unlikely to follow a truly grid-like pattern of movement under natural conditions. Furthermore, the discretisation imposed by the grid also distorts trajectories to an extent, as the agent does not move exactly in the direction indicated by the homing vector of a given position, but rather to the neighbouring grid intersection most closely aligned with it. Moreover, these distortions will also depend on the resolution of the grid. To illustrate this, one might imagine a perfectly continuous solution. Here, the agent will always move exactly according to the homing vector determined for its current location. This solution is equivalent to a grid-based solution of infinite resolution. If the grid resolution is lowered, more and more "nodes" on the original grid are lost. However, since each of these nodes might have sent the animal in a different direction, the trajectory taken by the animal across the same part of the environment will likely change as grid nodes are lost (see Fig 1, **a** vs. **B**). Nevertheless, while the trajectories for different grid resolutions might differ quite strongly, the overall effect on the reachability of locations is hard to estimate, since a given home location might gain new converging trajectories just as well as it might lose them.

To assess this systematically and avoid possible biases introduced by a specific grid resolution, homing trajectories were computed over a range of 8 *step sizes* between 10 and 80 world units per step. The trajectories computed with this technique often differed considerably depending on step size.

Furthermore, Basten & Mallot [4] name a divergence tolerance allowed for in their model to explain the surprisingly large catchment areas observed despite the expected limitations imposed by the constrained convergence of saddle points. Such a tolerance allows for successful homing even though the animal does not reach its exact home location, but passes by sufficiently close to it.

To assess how this tolerance affects homing success, we performed additional simulations

for one environment using 8 different *detection radii* between 10 and 80 world units.

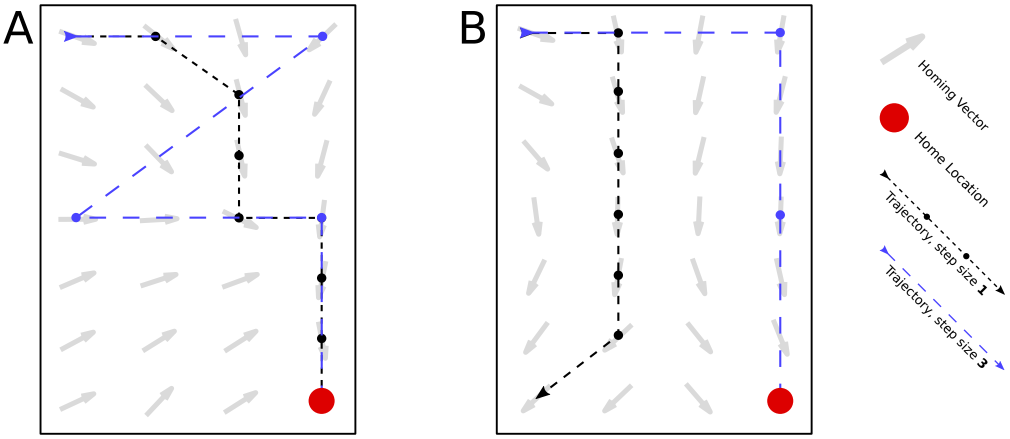

Fig 1. Distortion of trajectories due to different grid resolutions. To be able to compute homing trajectories efficiently, homing trajectories (*black* and *blue arrows*) were computed using a grid of equidistant locations (*black* and *blue* dots). However, the resolution of the grid can influence homing trajectories, leading to different paths across the same part of the environment (*blue* vs. *black* trajectory). These alternative paths might converge on a given home location (**A**), or they might diverge (**B**), leading to differences in the reachability of a location. Thus, to avoid possible biases induced by investigating homing only for a specific grid resolution, trajectories were computed systematically for different resolutions, leading to 8 step sizes between 10 and 80 world units per step. This was called the *step size* (between 1 and 8) of a given simulation. **A** and **B** depict trajectories using either the full grid (*black*), or every third grid node (*blue*), which corresponds to a step size of 1 or 3, respectively.

Finally, to determine the influence of the object density (i.e. the number of objects per area), additional simulations were performed to compare homing success in environments containing either 40, 80 or 120 objects.

Distribution of target types and catchment radii are robust across a wide range of different parameters

Our analysis shows that catchment radii increase, if the step size or detection radius is increased (see Fig 2). Thus, both a higher detection radius and a greater step size may increase the average reachability of home locations. However, changing either parameter does not solve the problem of limited convergence due to the prevalence of saddle points, as the proportion of different target types does not change (see Fig 3) over the whole range of parameters. Furthermore, the observed relation between target type and catchment radius also seems to be stable across all tested parameter combinations (see Fig 4). We conclude that the distribution of catchment area sizes and target types is

mostly robust against changes in the parameters used for the homing trial.

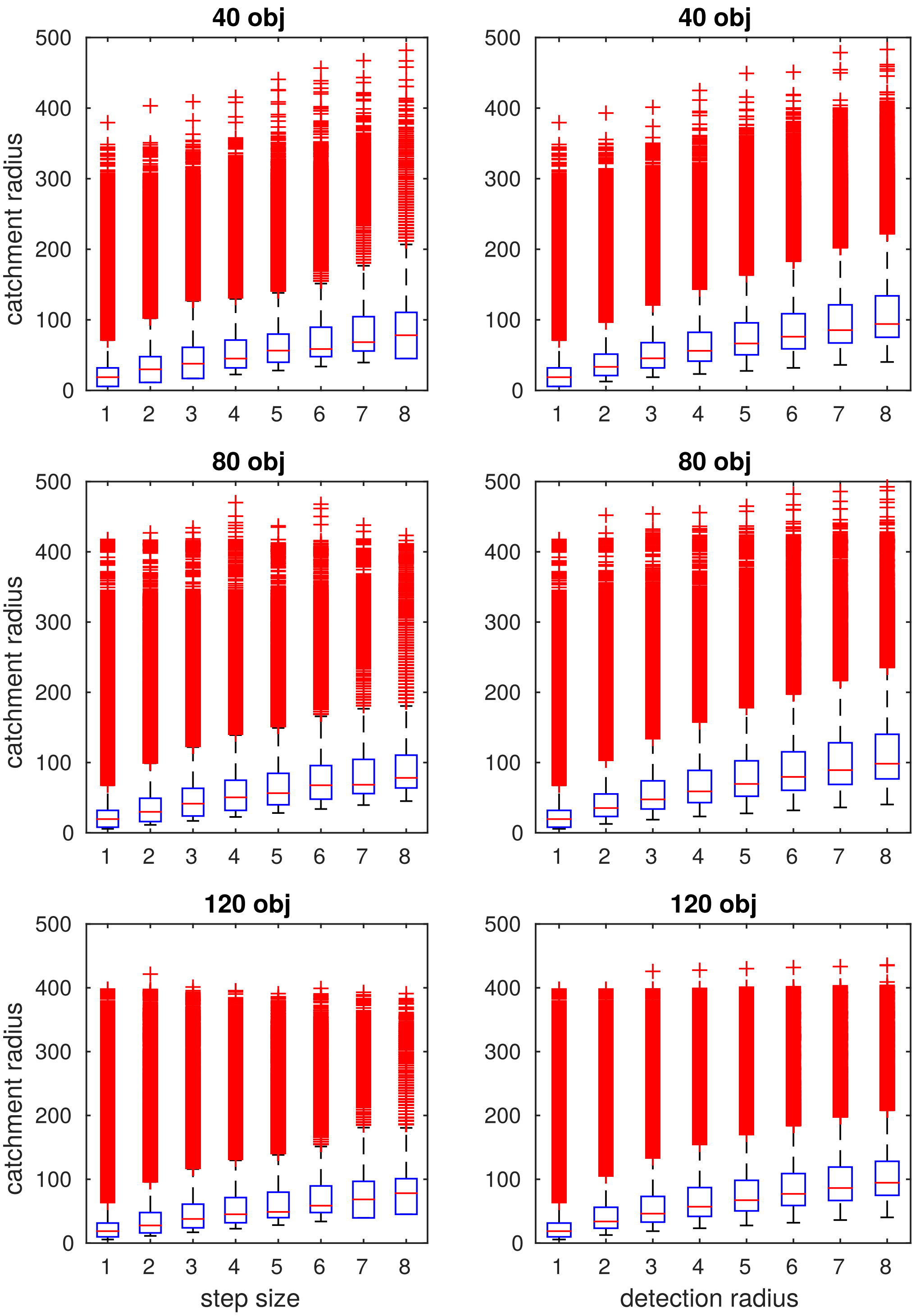

Fig 2. Distribution of catchment radii for homing runs of 8 different step sizes and 8 different detection radii in environments containing either 40, 80 or 120 objects.

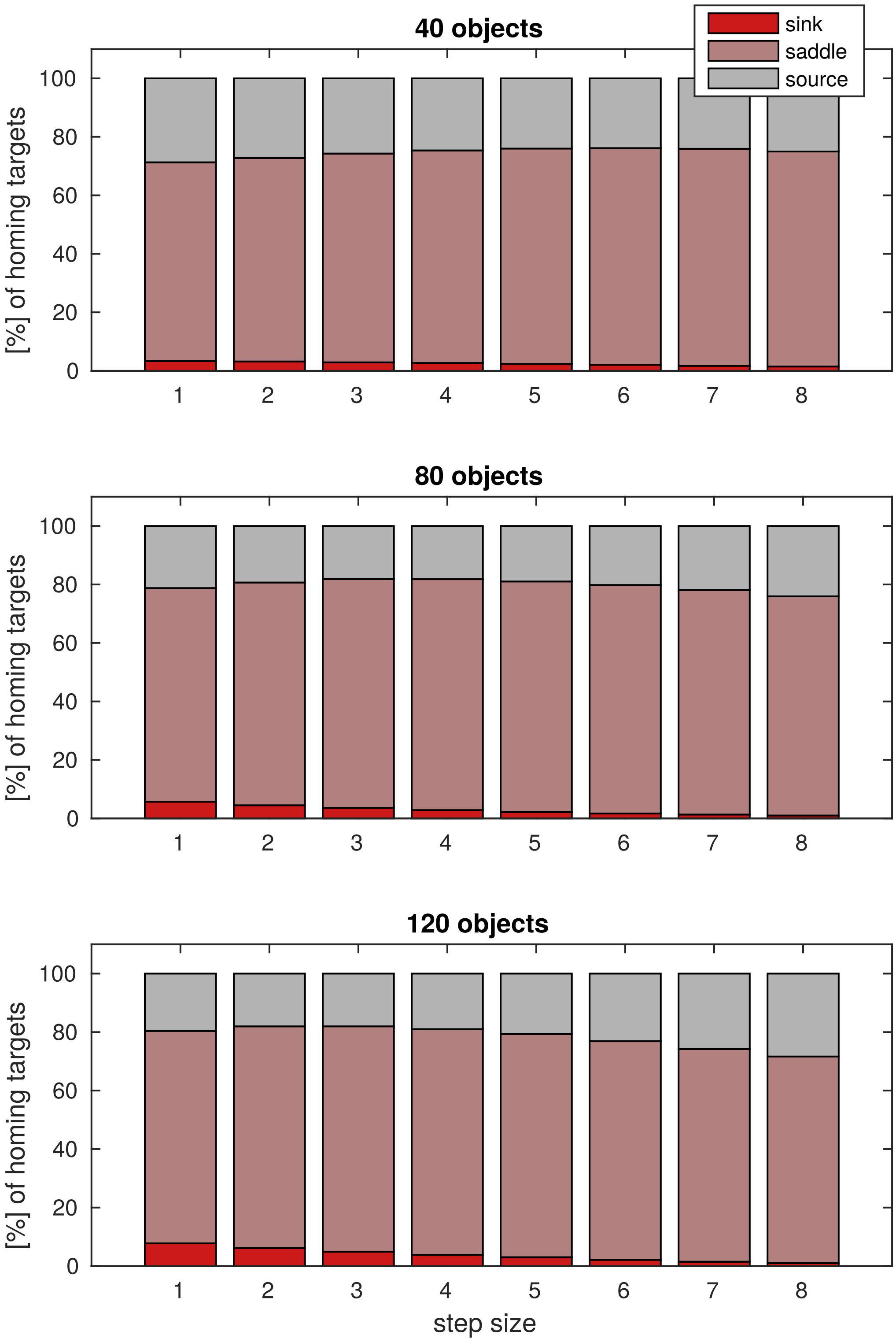

Fig 3. Proportion of target types for 8 different step sizes in environments containing either 40, 80 or 120 objects.

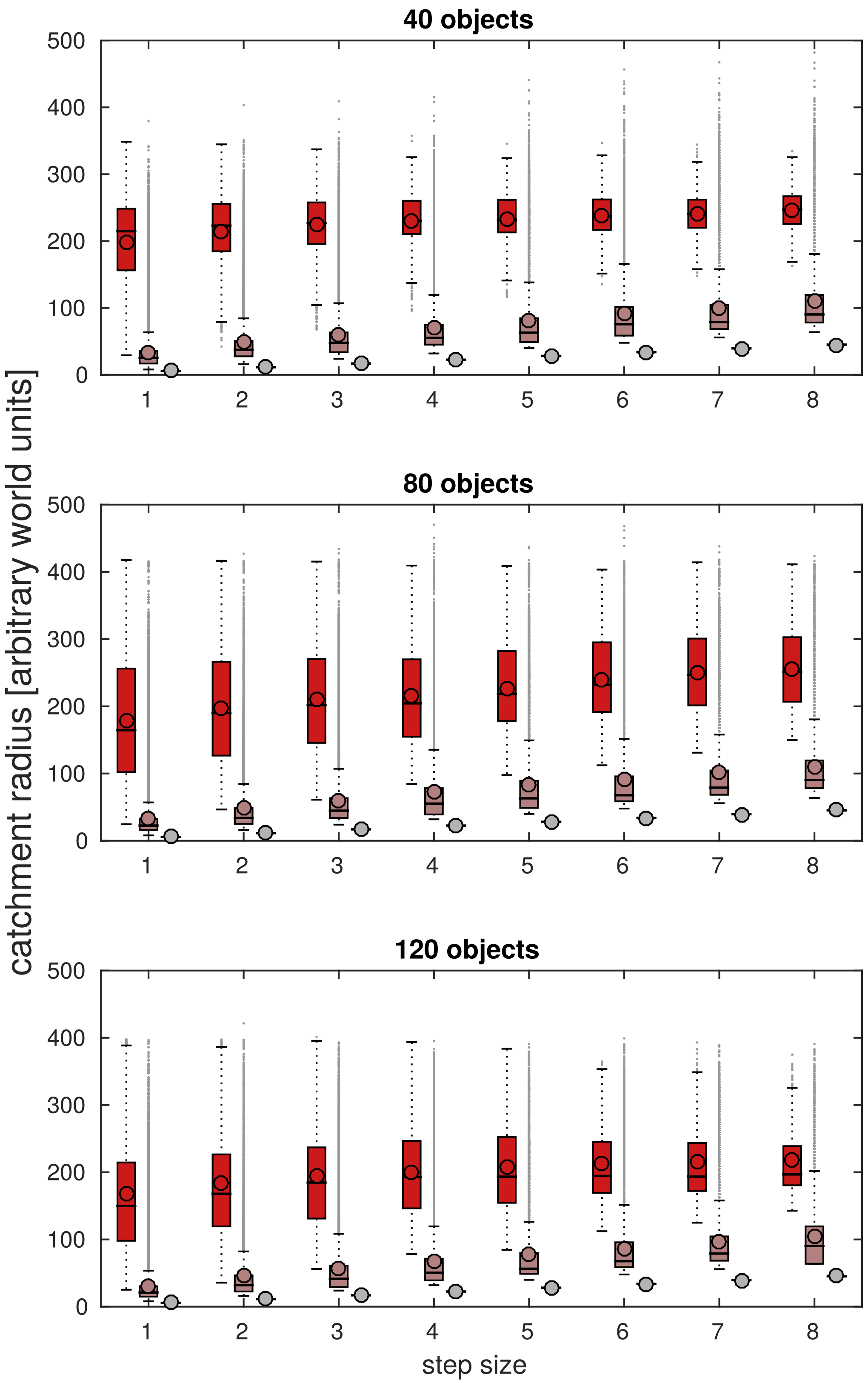
40 objects
80 objects
120 objects
catchment radius [arbitrary world units]
step size
0
100
200
300
400
500
1
2
3
4
5
6
7
8

Fig 4. Box plots of catchment radius distributions for all target types (sinks in deep red, saddle points in light red, sources in grey) for 8 different step sizes in environments containing either 40, 80 or 120 objects.

References

1. Mangan, M. (2011). Visual homing in field crickets and desert ants: a comparative behavioural and modelling study.
2. Mangan, M. and Webb, B. (2009). Modelling place memory in crickets. *Biological Cybernetics*, 101(4):307.
3. Murray, T. and Zeil, J. (2017). Quantifying navigational information: The catchment volumes of panoramic snapshots in outdoor scenes. *PloS one*, 12(10):e0187226.
4. Basten K, Mallot HA. Simulated visual homing in desert ant natural environments: efficiency of skyline cues. Biological Cybernetics. 2010;102(5):413–425. doi:10.1007/s00422-010-0375-9.
5. Stürzl W, Zeil J. Depth, contrast and view-based homing in outdoor scenes. Biological Cybernetics. 2007;96(5):519–531. doi:10.1007/s00422-007-0147-3.
6. Stürzl, W., Grixa, I., Mair, E., Narendra, A., and Zeil, J. (2015). Three-dimensional models of natural environments and the mapping of navigational information. *Journal of Comparative Physiology A*, 201(6):563–584.
